# Supplementary material for: Ground Reaction Forces and Impact Loading Among Runners with Different Acuity of Tibial Stress Injuries: Advanced Waveform Analysis for Running Mechanics
Source: Bioengineering (Basel). 2025 Jul 26;12(8):802. doi: 10.3390/bioengineering12080802 (PMC12383432; doi:10.3390/bioengineering12080802)

Supplementary Table 1. Running velocity and key spatiotemporal parameters among runners without injury, with tibial stress fracture (TSF) and medial tibial stress syndrome (MTSS). Values are means ± SD, covaried for age, sex and running velocity.

Variable	Not injured (n=33)	MTSS (n=12)	UL TSF (n=15)	BL TSF (n=6)	p	η^2
Velocity (km/h)	10.4 ± 1.1	9.4 ± 2.2	10.0 ± 1.7	9.8 ± 2.9	0.08	0.10
Cadence (step/min)	169 ± 9	166 ± 6	167 ± 13	167 ± 13	0.79	0.02
COM vertical displacement (mm)	9.2 ± 1.2	9.1 ± 1.4	9.3 ± 1.5	9.6 ± 1.5	0.86	0.01
Stride width (m)	0.08 ± 0.03	0.08 ± 0.04	0.07 ± 0.03	0.09 ± 0.04	0.88	0.01
Step length (m)						
Left	0.97 ± 0.11	0.94 ± 0.16	0.89 ± 0.21	0.86 ± 0.26	0.79	0.17
Right	0.98 ± 0.12	0.95 ± 0.17	0.89 ± 0.20	0.88 ± 0.27	0.81	0.01
Stance time (s)						
Left	0.24 ± 0.03	0.25 ± 0.04	0.25 ± 0.03	0.24 ± 0.03	0.89	0.01
Right	0.24 ± 0.03	0.25 ± 0.04	0.24 ± 0.04	0.25 ± 0.03	0.89	0.01

COM = center of mass

Supplemental Figure 1.

Peak impulse values during impact and active phases, during braking and propulsion and during medial and lateral excursion of stance

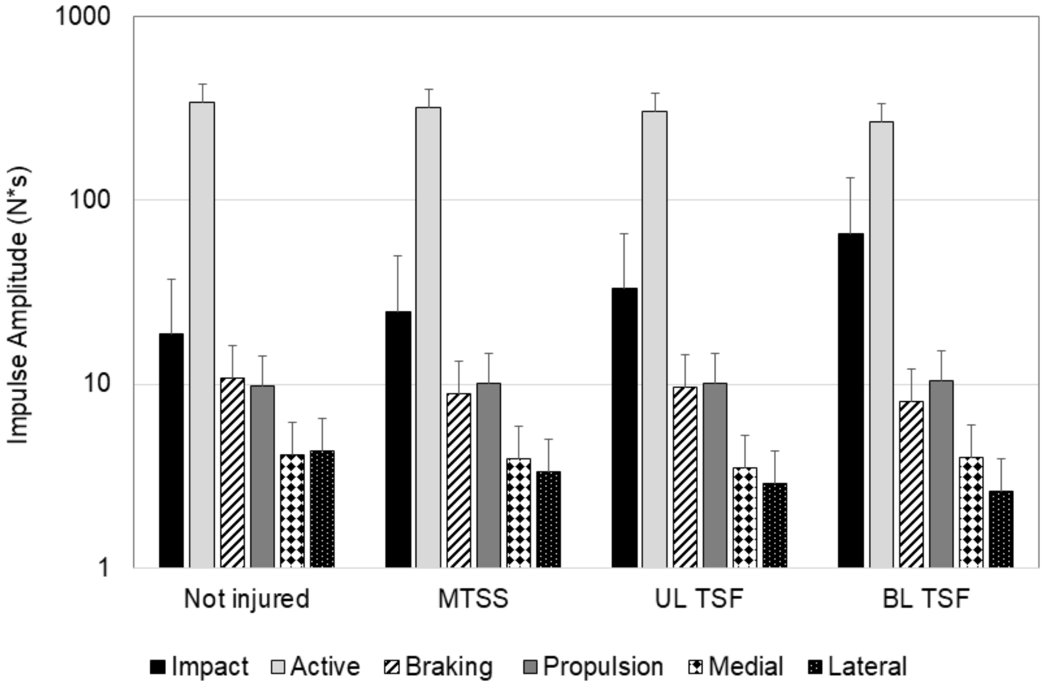

Supplement: Supplementary file 1 [file bioengineering-12-00802-s001.zip › bioengineering-3701232-supplementary.pdf]
